# The Relationship Between a Lifetime History of Sexual Victimization and Perinatal Depression: A Systematic Review and Meta-Analysis

**DOI:** 10.1177/15248380211021611

**Published:** 2021-06-16

**Authors:** Brooke N. Lombardi, Todd M. Jensen, Anna B. Parisi, Melissa Jenkins, Sarah E. Bledsoe

**Affiliations:** 1 School of Social Work, University of North Carolina at Chapel Hill, NC, USA

**Keywords:** sexual victimization, perinatal depression, survivor, meta-analysis, systematic review, trauma-informed care

## Abstract

**Background:**

The association between a lifetime history of sexual victimization and the well-being of women during the perinatal period has received increasing attention. However, research investigating this relationship has yet to be systematically reviewed or quantitatively synthesized.

**Aim:**

This systematic review and meta-analysis aims to calculate the pooled effect size estimate of the statistical association between a lifetime history of sexual victimization and perinatal depression (PND).

**Method:**

Four bibliographic databases were systematically searched, and reference harvesting was conducted to identify peer-reviewed articles that empirically examined associations between a lifetime history of sexual victimization and PND. A random effects model was used to ascertain an overall pooled effect size estimate in the form of an odds ratio and corresponding 95% confidence intervals (CIs). Subgroup analyses were also conducted to assess whether particular study features and sample characteristic (e.g., race and ethnicity) influenced the magnitude of effect size estimates.

**Results:**

This review included 36 studies, with 45 effect size estimates available for meta-analysis. Women with a lifetime history of sexual victimization had 51% greater odds of experiencing PND relative to women with no history of sexual victimization (*OR* = 1.51, 95% CI [1.35, 1.67]). Effect size estimates varied considerably according to the PND instrument used in each study and the racial/ethnic composition of each sample.

**Conclusion:**

Findings provide compelling evidence for an association between a lifetime history of sexual victimization and PND. Future research should focus on screening practices and interventions that identify and support survivors of sexual victimization perinatally.

The birth of a new baby is a transformative event in a woman’s life, often regarded as a joyful and fulfilling experience. Yet for some women, the perinatal period (i.e., conception through the first year postpartum) can be fraught with significant mental health challenges (Dossett, 2008). For example, up to 70% of women report depressive symptoms during pregnancy, with 10%–16% meeting the diagnostic criteria for major depressive disorder (MDD). Thirty-three percent of women diagnosed with MDD experience their first onset during pregnancy, and 40% experience their first depressive episode during the postpartum period (Becker et al., 2016). Overall, this research suggests that women may be especially vulnerable to depression during the perinatal period.

Perinatal depression (PND) represents a significant public health concern associated with a number of risks for mothers and children. For example, mothers who experience PND, on average, have higher rates of preeclampsia, preterm labor, cesarean delivery, and risk-taking activities (e.g., alcohol and drug abuse). Further, children whose mothers experience PND are more likely to fail to thrive, have low American Pediatric Gross Assessment Record scores (i.e., scores given to an infant minutes after birth that assess for physical condition), and suffer from emotional, behavioral, and/or cognitive delays (Bansil et al., 2010; Leung & Kaplan, 2009; Sanchez et al., 2013). These consequences underscore in urgent need to better understand the factors contributing to the development of PND.

Physicians, clinicians, and researchers alike have worked to identify biological, psychological, and psychosocial correlates of PND. Such correlates include imbalanced hormonal functioning, a family history of PND, insufficient social support, and low socioeconomic status, sexual orientation, and immigration status (Guintivano et al., 2018; Miller & LaRusso, 2011; Serati et al., 2016; Silverman & Loudon, 2010). However, a lifetime history of sexual victimization (i.e. one or more experiences of childhood and/or adulthood sexual abuse) has gained recognition over the last two decades as a significant predictor of PND (Aaron et al., 2015; Killian-Farrell et al., 2017; Shamblaw et al., 2019). The Centers for Disease Control and Prevention defines sexual victimization as any sexual act committed against a person without the freely given consent of that person (Smith et al., 2018). Sexual victimization is an issue that disproportionately affects women; nearly one in five women have been raped at some point in their lives, with the majority of women experiencing sexual abuse prior to the age of 25 (Black et al., 2011; Smith et al., 2018). Consequently, an emerging body of research has begun to explore associations between a lifetime history of sexual victimization and PND.

The prevalence of sexual victimization among women—particularly women entering the perinatal period—has significant implications for optimizing perinatal care. For example, the American College of Obstetricians and Gynecologists (ACOG) recommends that *all* women be screened for a history of sexual victimization; however, the low reported rates of sexual abuse among women receiving perinatal care indicate health care providers might not be asking routinely about a history of sexual victimization and/or asking in such a way that elicits disclosure (ACOG, 2006; Seng et al., 2008). A robust synthesis of research assessing a link between a lifetime history of sexual victimization and women’s health outcomes, particularly the presence or absence of PND, could bolster the case for consistent screening practices among practitioners who provide perinatal care.

An improved understanding of the relationship between sexual victimization and PND also has the potential to inform effective treatment services for women in the perinatal period (Benedict et al., 1999). Among pregnant women, a history of sexual victimization is associated with negative psychological sequelae such as increased levels of stress, anxiety, and suicidal ideations (Leeners et al., 2006). Sexual victimization may also evoke trauma-related symptoms, as the areas of a woman’s body that were violated during past incidences of sexual victimization are the same areas that are required to perform cervical checks, have a vaginal delivery, breastfeed an infant, and so on. Common trauma-related symptoms include hypervigilance and dissociation, which can impact women’s ability to seek and elicit care or respond to obstetric complications (Benedict et al., 1999; Hobbins, 2004). Moreover, the experiences of pregnancy, labor, and delivery may themselves trigger memories of prior sexual victimization, which can compound existing psychological complications and necessitate clinical follow-up and support (Benedict et al., 1999; Leeners et al., 2006). Consequently, increasing awareness of how sexual victimization and PND intersect could provide a foundation for improving treatment practices for women with a history of sexual victimization who are pregnant or in the perinatal period.

Although previous studies have examined the relationship between violence against women and PND, existing studies have largely evaluated multiple types of violence simultaneously, including physical, sexual, and emotional violence (Alvarez-Segura et al., 2014; Wu et al., 2012). Further, few studies have assessed the role of demographic characteristics, such as racial/ethnic identify, in shaping the relationship between a lifetime history of sexual victimization and PND. Moreover, we are not aware of any available publications that have systematically synthesized research regarding the link between PND and a lifetime history of sexual victimization specifically. To address this important gap in research, the primary goal of this systematic review and meta-analysis was to identify, summarize, and synthesize findings from studies investigating the association between a lifetime history of sexual victimization and PND. Although we acknowledge the growing literature exploring specific mechanisms linking a lifetime history of sexual victimization and PND (e.g., Choi et al., 2015; Choi et al., 2017; Mersky & Janczewski, 2018), our review focuses on the relatively larger share of available studies that emphasize direct associations between a lifetime history of sexual victimization and PND. We also conducted subgroup analyses to assess whether particular study features (i.e., other types of abuse, past depression, timing of sexual victimization, the instrument used to measure PND, bivariate vs. multivariate analyses, and the racial/ethnic composition of samples) were associated with the magnitude of effect size estimates.

## Method

### Eligibility Criteria

Our study search procedures conform to the Preferred Reporting Items for Systematic Review and Meta-Analyses guidelines (Liberati et al., 2009) and Cochrane Collaboration guidelines (Higgins & Green, 2019). Prior to the search, a study protocol was submitted through PROSPERO, an international prospective register for review protocols (registration # CRD42018097374).

Eligible studies were included in the review and meta-analysis if they met the following criteria: (a) peer-reviewed articles published in English, (b) quantitative studies that reported sufficient data to enable the calculation of effect sizes, (c) included sexual victimization as a distinct variable (rather than being combined with other forms of victimization), (d) quantitatively evaluated the association between sexual victimization and PND, and (e) included measures of depression between conception and one-year postpartum. Studies were excluded if they did not differentiate between different types of abuse (e.g., sexual, physical, or emotional) or focused exclusively on current intimate partner violence (IPV; i.e., a focus on victimization occurring concurrently with the perinatal period). Although IPV and sexual victimization are both known risk factors of depression (Biaggi et al., 2016; Trevillion et al., 2012), women experiencing IPV often experience multiple types of abuse (e.g., physical, emotional, and sexual; Trevillion et al., 2012) and live in active fear for their safety. Consequently, our choice to exclude studies focused on sexual victimization experienced in the context of IPV was intended to foreground associations between PND and the experience of a lifetime history of sexual victimization specifically.

### Search Strategy

The search strategy was developed in consultation with a health sciences reference librarian. A systematic, computerized literature search was conducted in the following four databases: PubMed, PsycINFO, Cumulative Index to Nursing and Allied Health Literature, and Global Health. The search was conducted from database inception to June 2020. Additional relevant publications were identified by manually searching the reference lists of articles identified in the present systematic review and meta-analysis.

Search terms were selected to identify empirical articles that examined the association between a history of sexual victimization and PND and consisted of the following terms and Boolean operators: *perinatal depression OR postpartum depression OR antenatal depression AND maternal outcomes OR obstetric outcomes AND pregnancy OR childbirth AND rape OR sexual violence OR sexual assault*.

### Study Selection

A team of three social science researchers developed a data extraction form prior to extracting data. This form was used to collect information related to study author, year, country of study recruitment, sample recruitment strategy, sample size, sample characteristics, measurement periods, timepoints of sexual victimization, instruments used to measure depression and sexual victimization, statistical analyses, and effect sizes. Two members of the research team used this form to independently extract data from five studies to determine interrater reliability. This process yielded a 95% agreement rate. Next, the remaining articles were divided between two members of the research team and for independent extraction. Any points of uncertainty were discussed, and data were managed using Covidence software (Veritas Health Innovation, 2018).

### Data Analysis

Following the systematic review process, we conducted a meta-analysis of effect sizes derived from studies assessing the association between a lifetime history of sexual victimization and PND. Given our expectation of significant between-study heterogeneity, the random effects model was favored over the fixed effects model (Littell et al., 2008). For additional context, random effects models assume that the true effect varies across study particulars, whereas fixed effects models assume that there is one true effect size and all studies come from the same population (Littell et al., 2008). As a sensitivity analysis, we reestimated the overall effect size using a fixed effects model to compare with results from the random effects model. In addition, the majority of included studies reported effect sizes using the odds ratio (*OR*) metric, resulting in our decision to estimate pooled *OR* estimates and corresponding 95% confidence intervals (CIs). For studies reporting other effect-size metrics, we converted effect sizes into the *OR* metric using the conventions and rationale outlined by Borenstein and colleagues (2009). The core rationale for metric conversion, at least in our case, is that the decision to use “conversions is often better than the alterative, which is to simply omit the studies that happened to use an alternative metric” (Borenstein et al., 2009, p. 46). As another sensitivity analysis, and consistent with best practices (Borenstein et al., 2009), we reestimated and reported the overall effect size without converted effect sizes.

Our meta-analytic models were estimated in Stata 16.0 (StataCorp, 2019) using the Dersimonian and Laird (1986) estimation method. Contour-enhanced funnel plots and Egger’s test were used to assess publication bias, and other potential sources of bias, with respect to effect size estimates across studies (Chaimani et al., 2014; Egger et al., 1997; Peters et al., 2008). To explore further the potential sources of between-study heterogeneity, and to assess the potential influence of study characteristics on average effect sizes, we employed a series of subgroup analyses. Five specific subgroup analyses were conducted to assess the potential influence of (a) analysis type (i.e., bivariate vs. multivariate), (b) use of covariates in the study models (i.e., none, other types of abuse, past depression, or both past depression and other types of abuse), (c) timing of sexual victimization (i.e., childhood, adulthood, lifetime, or unspecified), (d) the instrument used to measure PND, and (e) the racial/ethnic composition of study samples. Meta-regression was also used to assess the potential influence of study characteristics on effect size estimates; however, the model yielded no significant associations. Consistent with methodologists’ comments about the limitations of meta-regression, we believe our meta-regression model might have been burdened by low statistical power (Borenstein et al., 2009). As a result, we focus on the results from subgroup analyses.

## Results

### Study Selection and Characteristics

**Figure 1.**
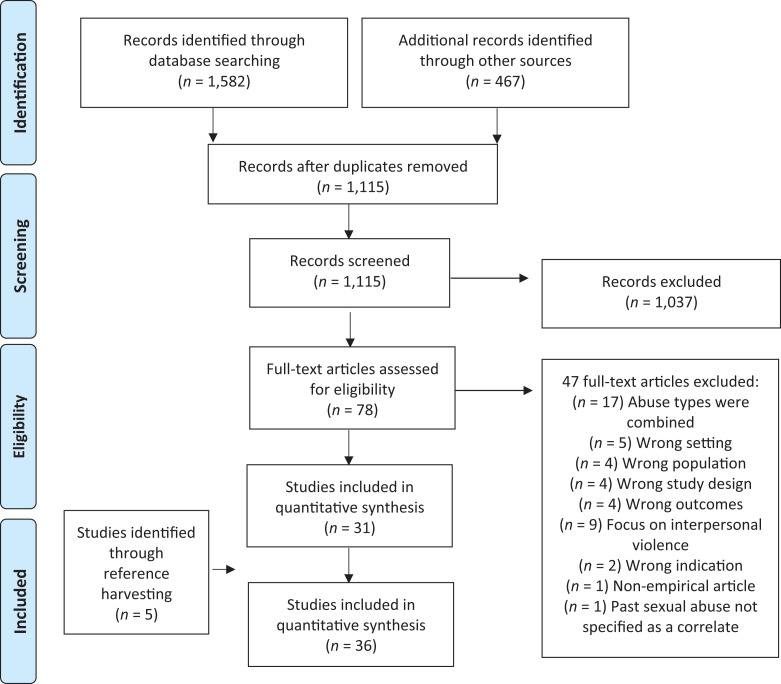
Flow chart for literature search and study selection process.

The article extraction process is presented in Figure 1. A total of 36 articles met the inclusion criteria and were included in the review. The most common reason for exclusion was the combination of sexual victimization with other abuse variables (e.g., physical, emotional, intimate partner victimization), which precluded evaluation of the impact of sexual victimization on PND independently.

Characteristics of the 36 included studies are displayed in Table 1. Sample sizes ranged from 44 to 53,065 participants. Studies were conducted across 39 different countries, most commonly in the United States (31.1%), and Canada (16.7%). A relatively similar number of studies in this review used longitudinal (*n* = 17) and cross-sectional designs (*n* = 19). The racial/ethnic composition of study samples varied. Over one third (39.2%) of included studies did not specify the racial or ethnic identities of their samples. Among the studies that did report this information, most evaluated samples of individuals who predominantly identified as White/Caucasian (27.6%), and the remaining studies evaluated samples that primarily identified as disparate non-White (14.1%), Black/African American (11.4%), or Asian (7.9%).

**Table 1.** Overview of Studies.

First Author and Year	Country	Recruitment Strategy and Design	Sample Size	Racial/Ethnic Characteristics	Timepoint of Sexual Victimization	Sexual Victimization Instrument	Depression Instrument	Odds Ratio (95% CI)
Aaron et al. (2015)	United States	Convenience; longitudinal	162	B/AA: 79%; O (W, A, H): 20.9%	Childhood (<16)	Self-report	CES-D	2.07 [1.01, 4.23]
Barrios et al. (2015)	Peru	Convenience; cross-sectional	1,521	Mestizo: 75.2%	Childhood (<18)	CPSAQ	PHQ-9	1.20 [0.73, 1.96]
Benedict et al. (1999)	Not specified	Convenience; cross-sectional	357	AA: 74%	Childhood (<18)	CPSAQ	CES-D	2.44 [1.12, 5.31]
Bonacquisti et al. (2014)	United States	Convenience; cross-sectional	163	B/AA: 79.1%; African: 1.8%; W: 2.5%; H: 9.8%; A: 1.2%; BI: 3.1%; O: 2.5%	Childhood	Self-report	CES-D	2.04 [1.07, 3.87]
Choi et al. (2015)	South Africa	Convenience; cross-sectional	84	Not specified	Childhood	CTQ	EPDS	2.61 [1.14, 5.97]
Chung et al. (2008)	United States	Convenience; longitudinal	1,476	AA: 71%; L: 17%; W9%; O: 3%	Childhood (<16)	Self-report	CES-D	1.69 [1.15, 2.48]
Cohen et al. (2002)	Canada	Convenience; cross-sectional	200	Not specified	Childhood (<14); Adulthood (>14)	CTS AAS	EPDS	0.29 [0.07, 1.29]
Dennis and Ross (2006)	Canada	Convenience sampling; longitudinal	634	W: 92%	Childhood	Self-report	EPDS	1.92 [1.11, 3.33]
Garabedian et al. (2011)	United States	Convenience; cross-sectional	5,380	W: 5.1%; O: 94.9%	Childhood	BRFS	Self-report	1.48 [1.26, 1.74]
Gilson and Lancaster (2008)	Australia	Convenience; longitudinal	79	Not specified	Childhood (<13)	Self-report	EPDS	7.57 [1.79, 32.08]
Gottfried et al. (2015)	Israel	Convenience; longitudinal	300	Not specified	Lifetime	SES	BDI	Antenatal: 1.13 [0.55, 2.34]; Postpartum: 2.36 [1.02, 5.47]
Jeong et al. (2013)	Korea	Convenience; cross-sectional	1,262	Not specified	Not specified	Not specified	EPDS	3.02 [1.49, 6.11]
Choi et al. (2018)	United Kingdom	Probability; longitudinal	1,016	Not specified	Childhood (< 18)	CTQ	DIS	Insufficient information to estimate effect size
Kendall-Tackett et al. (2013)	United States, Canada, United Kingdom, Australia, and New Zealand	Convenience; cross-sectional	6,410	From the United States: W; 91%; B: 2.5%; H/L: 67 (1.4%); A: 1.5%; NA: 0.6%	Lifetime	Self-report	PHQ-2	1.44 [1.27, 1.63]
Killian-Farrell et al. (2017)	United States	Convenience; longitudinal	210	W: 13.8%; L: 41.4%; AA: 40.5%; O: 4.3%	Before 13; after 13	TI	EPDS	Sex abuse before 13: 5.90 [2.38, 14.60]; Sex abuse after 13: 2.74 [1.01, 7.46]
Lang et al. (2006)	United States	Convenience; longitudinal	44	W: 61.4%; H: 18.2%; AA: 11.4%	Childhood	CTQ	BDI	4.37 [1.33, 14.38]
Leeners et al. (2014)	Germany	Convenience; cross-sectional	255	White: 99%	Childhood	Self-report	Self-report	1.2 [1.03, 1.30]
Lev-Wiesel and Daphna-Tekoah (2010)	Israel	Purposive; longitudinal	1,003	Jewish: 100%	Childhood (<14)	CSAS	CES-D	1.99 [1.32, 2.99]
Li et al. (2017)	China	Convenience; longitudinal	276	Han: 98.5%	Childhood	CTQ	EDPS	Antepartum depression: 5.05 [1.27, 20]; Postpartum depression: 2.42 [0.64, 9.18]
Littleton (2015)	United States	Convenience; cross-sectional	407	B: 53.6%; W: 36.9%; L: 3.2%; O: 2% Other 4.1%	Childhood (<14); adulthood (>14)	SES Self-report	CES-D	2.25 [1.57, 3.23]
Madigan et al. (2014)	Canada	Convenience; longitudinal	55	African/Caribbean or Caribbean: 49%; White: 29%; H: 13%; Portuguese: 5.5%; Canadian: 2%; A: 2%	Childhood	MCS	BDI	5.15 [1.40, 18.94]
Mahenge et al. (2018)	Tanzania	Convenience; cross-sectional	500	Not specified	Childhood	Self-report	PHQ-9	2.7 [1.35, 4.41]
Meltzer-Brody et al. (2013)	Netherlands	Purposive; longitudinal	682	Dutch: 100%	Childhood (<16); adulthood (>16)	CTI and ALES	EPDS	Sex abuse before 16: 3.43 [1.92, 6.13]; Sex abuse after 16: 4.05 [2.20, 7.45]
Nagl et al. (2017)	Germany	Convenience; cross-sectional	741	Not specified	Childhood (< 18)	CTQ	BDI	3.81 [1.28, 11.30]
Nelson et al. (2010)	United States	Convenience; cross-sectional	1,536	B: 91.8%	Childhood (<16)	Self-report	CES-D	2.35 [1.38, 4.00]
Plaza et al. (2012)	Spain	Convenience; cross-sectional	236	Not specified	Childhood (<18)	Self-report	EPDS	2.58 [1.12, 5.96]
Robertson-Blackmore et al. (2013)	Not specified	Convenience; longitudinal	374	W: 30.7%; B/AA: 48.7%; BI: 5.3%; H/L: 13.9%; O: 1.3%	Childhood	SCID	SCID	2.47 [1.27, 4.78]
Shah et al. (2011)	Pakistan and Canada	Stratified random; cross-sectional	384	Pakistani: 33.3%; W: 33.3%; Aboriginal: 33.3%	Not specified	Self-report	EPDS	Pakistani women 3.55 [1.28, 9.86]; Caucasian women 1.45 [0.36, 5.89]; Aboriginal women 3.11 [1.38, 7.02]
Sorbo et al. (2014)	Norway	Convenience; longitudinal	53,065	Not specified	Adult	AAS	EPDS	1.6 [1.4, 2.0]
J. Stevens et al. (2002)	United States	Convenience; cross-sectional	123	W: 77%; AA: 28%	Lifetime	K-SADS	BDI	Rape: 2.66 [1.29, 5.47]; Sexual abuse 2.02 [0.99, 4.15]
Tebeka et al. (2016)	United States	Probability; longitudinal	1,085	W: 65.5%; B: 12.7%; H: 15.7%; NA/A/Alaskan/ Hawaiian/Pacific Islander: 67 (6.1%)	Childhood	Self-report	AUDADIS-IV	3.07 [1.78, 5.28]
Verreault et al. (2014)	Canada	Convenience; longitudinal	364	W: 76.4%	Not specified	THQ	EPDS	1.74 [1.18, 2.57]
Wiemann et al. (1996)	United States	Convenience; cross-sectional	185	W: 33%; L: 29.7%; B: 33.5%; O: 3.8%	Childhood	Self-report	YSR	7.3 [1.7, 30.9]
Yampolsky et al. (2010)	Israel	Random; cross-sectional	1,835	Not specified	Not specified	SES	CES-D	1.08 [0.82, 1.43]
Zvara et al. (2017)	United States	Probability; longitudinal	204	Not specified	Childhood (<14)	THQ	BSI	1.20 [0.73, 1.97]
Yang et al. (2015)	China	Convenience; cross-sectional	5,391	Han: 100%	Childhood	Not specified	CICI	1.3 [1.0, 1.7]

*Note*. A = Asian; AAS = Abuse Assessment Screen; ALES = Adverse Life Events Scale; AUDADIS-IV = National Institute on Alcohol Abuse and Alcoholism’s Alcohol Use Disorder and Associated Disabilities Interview Schedule-*DSM*-IV; B/AA = Black/African American; BDI = Beck Depression Inventory; BI = Biracial; BRFS = Behavioral Risk Factor Surveillance Survey Questionnaire; BSI = Brief Symptoms Inventory; CES-D = Center for Epidemiological Studies-Depression Scale; CICI = Composite International Diagnostic Interview; CPSAQ = Childhood Physical and Sexual Abuse Questionnaire; CSAS = Child Sexual Assault Scale; CTI = Childhood Trauma Inventory; CTQ = Childhood Trauma Questionnaire; EPDS = Edinburgh Postnatal Depression Scale; H = Hispanic; K-SADS = Kiddie Schedule for Affective Disorders and Schizophrenia; L = Latina; MCS = Maltreatment Classification Scale; NA = Native American; O = Other; PHQ = Patient Health Questionnaire; SCID = Structured Clinical Interview for *DSM*-IV; SES = Sexual Experiences Survey; THQ = Trauma History Questionnaire; TI = Trauma Index; W = White; YSR = Youth Self-Report Scale.

### Sexual Victimization Measures

As displayed in Table 1, 14 assessment tools were used across 36 studies to assess for a lifetime history of sexual victimization. The most frequently cited assessment tools were the Childhood Trauma Questionnaire (Bernstein et al., 1994), which was used by six studies, and the Sexual Experiences Survey (Koss & Gidycz, 1985), which was used by four studies. Notably, two studies did not specify how a sexual victimization history was assessed. However, the vast majority of included studies relied on ad hoc, self-report measures to determine whether participants had a sexual victimization history.

### PND Measures

Ten separate assessment tools were used to assess for PND across the 36 included studies (refer to Table 1). The majority of studies in this review relied on the Edinburgh Postnatal Depression Scale (Murray & Cox, 1990). Other commonly used measures included the Center for Epidemiological Studies–Depression Scale (CES-D; Radloff, 1977), which was used by eight studies, and the Beck Depression Inventory (BDI; Beck & Gable, 2001), which was used by five studies. Only two studies used ad hoc measures to assess for depression.

### Overall Effect Size, Heterogeneity, and Sensitivity Analyses

**Figure 2.**
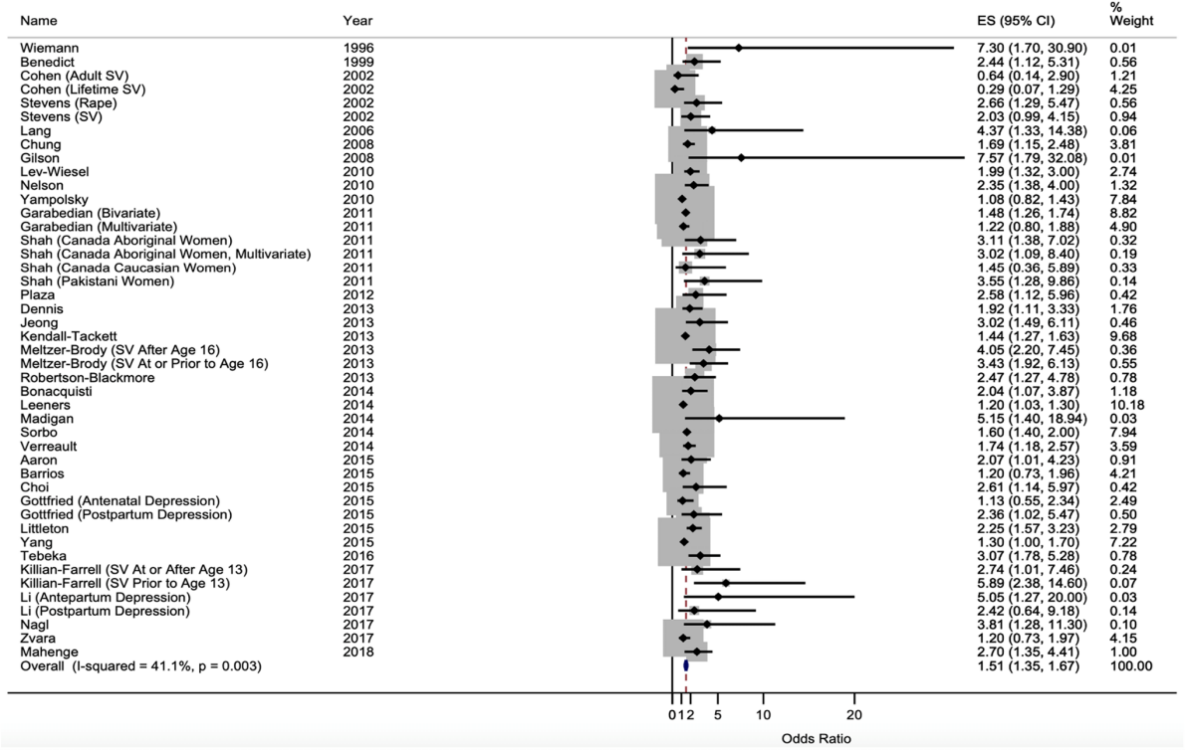
Forest plot of the association between past sexual victimization and perinatal depression.

Results from the meta-analysis of the overall effect size indicated a significant average effect size (*OR* = 1.51, 95% CI [1.35, 1.67]), such that a lifetime history of sexual victimization was associated with a 51% increase in the odds of experiencing PND. Figure 2 displays a forest plot associated with the included effect size estimates and the overall effect size. Results also indicated significant between-study heterogeneity as indicated by the *I*-squared statistic (*I* ^2^ = 41.1%, *p* = .003), which represents the percentage of the variation in effect estimates that is due to heterogeneity as opposed to sampling error or chance (Littell et al., 2008). Thus, our use of the random effects model was likely warranted (Table 2).

**Table 2.** Summary of Critical Findings.

Experience(s) of sexual victimization increase the odds of perinatal depression (*OR* = 1.51, 95% CI [1.35, 1.67]), even when controlling for other types of abuse, a history of depression, and other types of abuse and a history of depression.
When compared to other racial groups, Black/African American women with lifetime histories of sexual victimization were found to have the highest odds experiencing perinatal depression. This may be due to racial and socioeconomic inequities Black/African American women experience.
Average effect size estimates differed by the instrument used to measure perinatal depression. The largest effect sizes were observed among instruments that were consistent with the criteria in the *DSM*-IV.
Effect size estimates were significant for women who experienced sexual victimization in childhood, adulthood, and across the life course.

For comparison, the reestimation of the overall effect size using a fixed effects model yielded the following average effect size: *OR* = 1.37 (95% CI [1.29, 1.44]). In terms of the potential impact of including studies with converted effect-size metrics, the reestimation of the overall effect size with the omission of converted effect sizes yielded the following average effect size: *OR* = 1.44 (95% CI [1.24, 1.64]). Taken together, results from these sensitivity analyses suggest that the pooled *OR* estimate might not be overly burdened by our decision to use a random effects model and incorporate converted effect sizes.

### Subgroup Analyses

Table 3 displays results associated with subgroup analyses, with reference to the overall effect size. Turning to analysis type, effect sizes from bivariate analyses (*n* = 19) yielded a significant average effect (*OR* = 1.69, 95% CI [1.47, 1.92]), such that a lifetime history of sexual victimization was associated with a 69% increase in the odds of experiencing PND. Effect sizes from multivariate analyses (*n* = 26) also yielded a significant average effect (*OR* = 1.36, 95% CI [1.17, 1.56]), although this effect was relatively lower in magnitude. Significant between-study heterogeneity was apparent among effect sizes derived from multivariate analyses (*I* ^2^ = 41%, *p* = .02).

**Table 3.** Subgroup Analyses.

Outcome	Number of Effect Sizes	*OR*	95% CI	Weight (%)	*I^2^* (%)	*p*-Value for Test of Heterogeneity
Overall	45	1.51	[1.35, 1.67]	100	41.1	.003
Type of analysis						
Bivariate	19	1.69	[1.47, 1.92]	33.8	16.3	.255
Multivariate	26	1.36	[1.17, 1.56]	66.2	40.7	.017
Covariates						
None	24	1.63	[1.44, 1.82]	40.4	10.3	.317
Other abuse^a^	12	1.30	[0.89, 1.70]	26.8	48.5	.030
Past depression	4	1.34	[1.04, 1.63]	13.4	0.0	.573
Both past depression and other abuse	5	1.57	[1.10, 2.05]	19.4	68.6	.013
Timing of sexual victimization						
Childhood	27	1.53	[1.33, 1.73]	56.2	28.7	.083
Adulthood	4	1.72	[0.71, 2.73]	9.8	47.3	.128
Lifetime	7	1.47	[0.89, 2.05]	21.2	70.1	.003
Unspecified	7	1.62	[1.01, 2.23]	12.8	33.5	.172
Perinatal depression measure						
EPDS	19	1.86	[1.33, 2.39]	22.4	48.2	.010
CES-D	8	1.81	[1.34, 2.28]	21.2	53.7	.034
BDI-II	7	1.68	[1.01, 2.37]	4.7	0.0	.530
PHQ	3	1.45	[1.05, 1.85]	14.8	37.3	.203
*DSM*	3	2.02	[0.84, 3.21]	8.8	61.8	.073
Other measures^b^	5	1.28	[1.13, 1.43]	28.1	14.6	.321
Racial/ethnic composition of sample						
Asian	4	1.35	[1.01, 1.70]	7.85	0.0	.403
Majority Black/African American	7	2.03	[1.61, 2.45]	11.35	0.0	.928
Majority non-White^c^	6	1.45	[1.23, 1.67]	14.07	0.0	.453
Majority White/Caucasian	8	1.48	[1.21, 1.74]	27.56	51.0	.047
Not specified	20	1.49	[1.18, 1.84]	39.17	52.0	.004

*Note*. BDI = Beck Depression Inventory, Second Edition; CES-D = Center for Epidemiological Studies Depression Scale; CI = confidence interval; *DSM* = Diagnostic and Statistical Manual of Mental Disorders Criteria; EPDS = Edinburgh Postnatal Depression Scale; *OR* = odds ratio; PHQ = Patient Health Questionnaire.

^a^ Other abuse indicates that the effect size was estimated in the original study in the presence of covariates representing other forms of past abuse (e.g., physical abuse, emotional abuse).

^b^ Other measures of perinatal depression include self-report instruments, the Youth Self-Report Scale, and the Brief Symptoms Inventory.

^c^ Majority non-White samples included the following: one effect size estimated with majority Mexican American and Black/African American sample; one effect size estimated with majority African, Caribbean descent, and Hispanic sample; two effect sizes estimated with majority Black/African American and Latinx sample; and two effect sizes estimated with an unspecified majority non-White sample.

Studies also differed in terms of model covariates present when estimating the association between a lifetime history of sexual victimization and PND. Twenty-four effect size estimates were derived from models with no covariates, yielding a significant average effect size (*OR* = 1.63, 95% CI [1.44, 1.82]). Similar but slightly attenuated average effect sizes were estimated from studies using models that included (a) other types of abuse (12 effect size estimates; *OR* = 1.30, 95% CI [0.89, 1.70]), (b) past depression (four effect size estimates; *OR* = 1.34, 95% CI [1.04, 1.63]), and (c) both past depression and other types of abuse (five effect size estimates; *OR* = 1.57, 95% CI [1.10, 2.05]). Note that the average effect size derived from models in which both past depression and other types of abuse were included as model covariates represents a relatively robust assessment of the association between a lifetime history of sexual victimization and PND. Between-study heterogeneity was most pronounced among studies with other-abuse covariates (*I* ^2^ = 49%, *p* = .03) and studies with covariates for both other types of abuse and past depression (*I* ^2^ = 69%, *p* = .01).

Results from subgroup analyses also suggested slight variation in average effect sizes with respect to the timing of sexual victimization. Twenty-seven effect sizes from studies focused on childhood sexual victimization yielded a significant average effect size (*OR* = 1.53, 95% CI [1.33, 1.73]). Similar average effect sizes were estimated from studies focused on (a) adulthood sexual victimization (four effect size estimates; *OR* = 1.72, 95% CI [0.71, 2.73]), (b) lifetime sexual victimization (seven effect size estimates; *OR* = 1.47, 95% CI [0.89, 2.05]), and (c) unspecified timing of sexual victimization (seven effect sizes estimates; *OR* = 1.62, 95% CI [1.01, 2.23]), although the average effect sizes in these groups yielded wider CIs. Notably, between-study heterogeneity was most pronounced among studies focused on lifetime sexual victimization (*I* ^2^ = 70%, *p* = .003).

Average effect size estimates also differed across studies with respect to the instrument used to measure PND. Nineteen effect sizes from studies using the EPDS instrument yielded a significant average effect size (*OR* = 1.86, 95% CI [1.33, 2.39]); eight effect sizes from studies using the CES-D instrument yielded a significant average effect size (*OR* = 1.81, 95% CI [1.34, 2.28]); three effect sizes from studies using the PHQ instrument yielded a significant average effect size (*OR* = 1.45, 95% CI [1.05, 1.85]); three effect sizes from studies using instruments consistent with diagnostic criteria from the *Diagnostic and Statistical Manual of Mental Disorders* (*DSM*-IV) yielded a relatively larger average effect size (*OR* = 2.02, 95% CI [0.84, 3.21]); and five effect sizes from studies using other measures of PND yielded a significant, and relatively smaller, average effect size (*OR* = 1.28, 95% CI [1.13, 1.43]). The remaining seven effect sizes from studies using the BDI-II yielded a significant average effect size (*OR* = 1.68, 95% CI [1.01, 2.37]). Between-study heterogeneity was most pronounced among studies using the EPDS, CES-D, and *DSM*-related instruments (*I* ^2^ = 48%, *p* = .01, *I* ^2^ = 54%, *p* = .03, and *I* ^2^ = 62%, *p* = .07, respectively).

Subgroup analyses also yielded fairly similar average effect size estimates with respect to the racial/ethnic composition of study samples. Four effect sizes were estimated from studies focused on Asian samples, with a significant average effect size (*OR* = 1.35, 95% CI [1.01, 1.70]). Six effect sizes were estimated from studies focused on majority non-White samples (i.e., one effect size was estimated with a majority Mexican American and Black/African American sample; one effect size was estimated with a majority African, Caribbean descent, and Hispanic sample; two effect sizes were estimated with majority Black/African American and Latinx samples; and two effect sizes were estimated with unspecified majority non-White samples). This cluster of effect sizes yielded a significant average effect size (*OR* = 1.45, 95% CI [1.23, 1.67]). Eight effect sizes were estimated from studies focused on majority White/Caucasian samples, with a significant average effect size (*OR* = 1.48, 95% CI [1.21, 1.74]). Twenty effect sizes were estimated from studies focused on samples with unspecified racial/ethnic compositions, yielding a similar average effect size as that yielded from majority White/Caucasian samples (*OR* = 1.49, 95% CI [1.18, 1.84]). The remaining seven effect sizes were estimated from studies focused on majority Black/African American samples, with a significant and larger average effect size (*OR* = 2.03, 95% CI [1.61, 2.45]). Between-study heterogeneity was significant among studies using majority White/Caucasian samples (*I* ^2^ = 51%, *p* = .05) and samples with unspecified racial/ethnic compositions (*I* ^2^ = 52%, *p* = .01).

### Assessment of Bias

**Figure 3.**
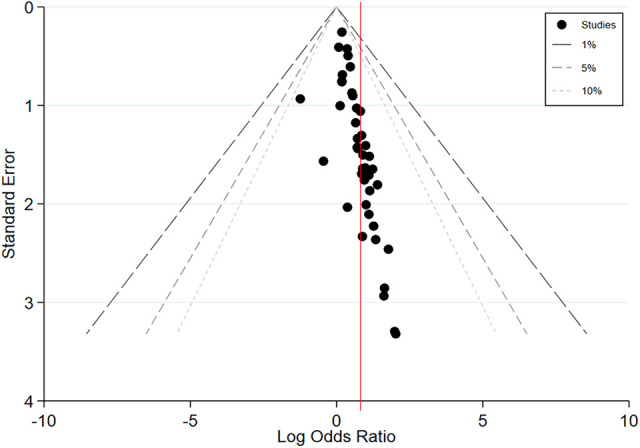
Contour-enhanced funnel plot of all effect size estimates.

Results from Egger’s test indicated significant asymmetry with respect to effect size estimates and corresponding standard errors, which signaled the potential for publication bias (i.e., bias resulting from studies with null findings not being submitted or accepted for publication, or bias incurred by inflated effect size estimates in studies with relatively smaller samples; Chaimani et al., 2014; Egger et al., 1997). Figure 3 displays a contour-enhanced funnel plot, which aids in distinguishing between publication bias and other potential causes of asymmetry or bias (Chaimani et al., 2014). As shown in the figure, effect size estimates from studies of all sizes clustered within the statistically nonsignificant region of the plot, suggesting that asymmetry could be caused by other forms of bias in addition to publication bias (Chaimani et al., 2014). Consequently, conclusions related to average effect size estimates should be drawn with some caution.

## Discussion

Although prior reviews (Alvarez-Segura et al., 2014) and meta-analyses (Wu et al., 2012) have examined the associations between a history of violence (e.g., past sexual, emotional, physical, and intimate partner violence) and PND, this review evaluated the impact of sexual victimization independently from other types of violence. Our review of 36 studies yielded substantial evidence for a significant association between a lifetime history of sexual victimization and PND. Specifically, on average, we found that the women who had a history of sexual victimization had 51% greater odds of experiencing PND relative to women without this history.

### Subgroup Analyses

The number and variety of studies included in this review enabled us to conduct five specific subgroup analyses to assess possible sources of effect-size variation. These included (a) analysis type, (b) use of covariates in the study models, (c) timing of sexual victimization, (d) the instrument used to measure PND, and (e) the racial/ethnic composition of study samples.

### Types of Analysis and Covariates

We found that the overall pooled effect size between sexual victimization and PND remained significant whether they were derived from bivariate or multivariate analyses. Among studies conducting multivariate analyses, there was significant variation with respect to the number and nature of covariates included in the analyses. Nevertheless, significant effects were observed even when models controlled for other types of abuse, past depression, and both past depression *and* other types of abuse. These findings bolster the case that a lifetime history of sexual victimization increases the odds for experiencing PND, above and beyond the influence other covariates.

### PND Measure

We found that average effect size estimates differed across studies with respect to the instrument used to measure PND. The largest effect sizes were observed for studies using the EPDS or criteria consistent with the *DSM*, and the smallest effect sizes observed among studies using other measures, which included self-report instruments, the Youth Self-Report Scale, and the Brief Symptoms Inventory. The consistency of positive findings across studies using different assessment measures supports the significance of the association between sexual victimization and PND. However, the diminished strength of the effect sizes observed among studies using self-report measures is notable. In order to most accurately assess for depression, future studies should rely on validated measures or those derived from clinical diagnostic criteria.

#### Timing of sexual victimization

The majority of included studies in the present review (*n* = 27) evaluated childhood sexual victimization. The remaining studies evaluated adulthood sexual victimization (*n* = 4), a lifetime history of sexual victimization (*n* = 7), or did not specify a period of time in which victimization was evaluated (*n* = 7). Notably, effect sizes with overlapping CIs were observed for all four groups, suggesting that sexual victimization could be a significant risk factor of subsequent PND regardless of the age at which victimization first occurs. However, given the slight variation in average effect sizes that were observed with respect to the timing of sexual victimization, there is a need for further research to explore variation in the impact of sexual victimization at different periods over the life course.

The predominance of studies focused on childhood sexual victimization observed in the present review reflects an extensive body of literature documenting the elevated prevalence and significant consequences of child sexual victimization specifically. According to the 2015 National Intimate Partner and Sexual Violence Survey (Smith et al., 2018), 43.6% of women have experienced some form of sexual victimization in their lifetime. However, although the majority (81.3%) of women who have experienced completed or attempted rape report that this victimization initially occurred prior to the age of 18, a significant number of women experience victimization for the first time as adults. Further, childhood sexual victimization is a known risk factor of sexual revictimization in adulthood (Scoglio et al., 2021; Tapia, 2014; Ullman et al., 2009). Consequently, future studies could benefit from determining whether accumulated experiences of sexual victimization across the life span influence the women’s likelihood of experiencing PND.

### Diversity

As indicated in Table 3, nearly half (44%) of the effect sizes included in the meta-analysis were yielded from studies that did not provide detailed information about the racial/ethnic composition of their samples. This omission reflects a significant shortcoming of the literature focused on associations between a lifetime history of sexual victimization and PND. Moving forward, researchers should be vigilant in their efforts to describe their samples with sufficient detail, ensuring racial data are disaggregated so that readers can better ascertain how study findings might be generalized to different populations. Further, it is worth noting that among studies in which racial/ethnic information about samples was offered, a sizable portion of them focused on samples comprised of Asian participants, majority Black/African American participants, and varying sets of majority non-White/Caucasian participants. Future research should continue foregrounding potential racial/ethnic differences with respect to the association between a lifetime history of sexual victimization and PND.

The average effect size estimate for the association between a lifetime history of sexual victimization and PND was observed to be highest from majority Black/African American samples. These results suggest the potential value of developing tailored screenings and interventions that consider complex psychosocial risk factors, many of which can vary with respect to one’s racial/ethnic identity. It is well-documented that Black/African American women encounter significant economic barriers (O’Hara & McCabe, 2013) and racial discrimination (Lara-Cinisomo et al., 2018). These experiences reflect the pervasive institutional and structural racism experienced by Black/African American individuals, which have been found to hinder the ability of this population to reliably access needed supports following experiences of sexual victimization (Ullman & Lorenz, 2020; White, 1999).

Prior studies have found that Black/African American mothers experience higher levels of stress throughout their lifetime, creating a “weathering” effect due to the ongoing consequences of socioeconomic disadvantages associated with discriminatory racial policies and practices (Deal et al., 2014). Given these findings, incorporating principles of community empowerment, resiliency, and cultural competency into service coordination and perinatal care interventions may be warranted in order to improve stress levels among Black/African American mothers who have experienced sexual victimization (Willis et al., 2014). Further research should focus on embedding a culturally relevant understanding of perinatal practice with consideration of women’s prior trauma exposure, including training in integrated frameworks such as a life course approach.

### Strengths and Limitations

To the best of our knowledge, this review and meta-analysis is the first to specifically examine the association between a lifetime history of sexual victimization and PND. Our meta-analytic approach yielded important insights related to how various methodological and substantive choices across studies (e.g., timing of sexual victimization, past abuse, past depression, depression instrument, and the racial/ethnic composition of the sample) impacted the overall effect size estimates.

Any conclusions or implications drawn from our findings should be tempered by some limitations associated with our review and meta-analysis. First, more than one third (35.6%) of included studies relied on nonvalidated assessment tools to identify survivors of sexual victimization, some of which comprised a single question asking participants whether they had been raped or forced into unwanted sexual activity. However, prior studies have found that experiences of sexual victimization are commonly underreported, particularly when assessed using a single screening item or through questions using terms such as “rape,” given that many survivors may not consider their experiences to be rape (Koss, 1993; Lemaire et al., 2016; Testa et al., 2004). Thus, we recommend that multiple behaviorally specific questions be used to assess for a sexual victimization history (Palmieri & Valentine, 2021; Testa et al., 2004). Second, our study excluded women who experienced sexual violence during the perinatal period within the context of an intimate partner relationship. Consequently, IPV-related violence remains an issue not reflected in our meta-analytic findings. Third, this study included race/ethnicity in the subgroup analyses, as this was the only demographic variable reported by a sufficient number of studies to enable meaningful subgroup analysis. However, future studies examining associations between PND and sexual victimization would benefit from the examination of other demographic characteristics (e.g. sexual orientation, socioeconomic status, immigration status) in order to (a) improve our understanding of how such characteristics may influence the relationship between a lifetime history of sexual victimization and PND and (b) enable evaluation of such characteristics in future syntheses of this body of evidence. Fourth, the articles included in this article represent a heterogeneous sample with a wide variety of cultural differences. Thus, the results may not be generalizable to all populations of people with a history of sexual victimization. Fifth, gray (i.e., unpublished) literature was purposefully excluded from this review, as we sought to only include articles that had gone through the rigorous process of peer review. As a result, our review does not include unpublished study findings that could influence the magnitude of the overall pooled effect size estimate presented.

### Policy, Practice, and Research Implications

Sexual victimization has been recognized by professional medical organizations as a major public health issue that requires the attention of health care professionals (ACOG, 2019; American Medical Association [AMA], 2008; Hillard, 2019). We urge all perinatal health practitioners to assess for a history of sexual victimization and provide resources and services to many women whose needs might have previously been overlooked. These services are particularly important when survivors are women of racial/ethnic minorities, as the impact of economic inequities and stigma associated with racial discrimination can intensify the negative effects of sexual victimization, reflecting a need for trauma-informed services that holistically address these issues (Willis et al., 2014). Based on our findings, we offer suggestions for future research, policy, and practice needs that are summarized and described in detail below.

The AMA (2008) and the ACOG (2019) recommend routine screening for all women with appropriate and timely care when sexual violence has been identified. However, recommendations for *routine*, *appropriate*, or *timely* screening are not defined or standardized. This study reaffirms the importance of the ACOG’s recommendations and highlights the need for health care professionals to be trained in routine screening practices for sexual victimization, trauma-informed obstetric care, and increased institutional support for providers providing care for survivors (e.g., training, continuing education, clear policies and procedures, and how to access insurance reimbursement for routine screening; Rose et al., 2011; Stevens & Sheaffer, 2007). Trauma-informed care is an integral component to obstetric care for all women and has been found to bring a balance of power in provider–patient relationships, establish trusting relationships, and reduce symptoms of PTSD and depression (Reeves, 2015; Seng et al., 2011; N. R. Stevens et al., 2019) Further, screening recommendations such as *routine*, *appropriate*, and *timely* must be standardized in order to improve the clarity and utility of such recommendations for health care providers.

A wide range of psychosocial and health-related consequences are associated with childhood sexual victimization (Hailes et al., 2019); however, results from the present study indicate that child and adult sexual victimization are associated with depression during the perinatal period. These findings underscore the need for recommendations that better assess for a history of sexual victimization and intervene when such history is disclosed. As such, it is essential that providers carefully assess for a history of sexual victimization occurring over the course of a lifetime rather than assessing for such a history exclusively during childhood. The findings from this study also highlight the need for the implementation of trauma-informed perinatal care. Although some women will disclose experiences of sexual victimization, the majority of survivors do not share such information even when asked directly (Stevens & Sheaffer, 2007). As such, perinatal care must be trauma-informed regardless of whether the patient has disclosed a history of victimization. Findings from qualitative studies could be especially informative on this front, as research of this sort provides an avenue through which survivors can narrate the contours and nuances of their experiences, highlight self-identified strengths, and emphasize perceived service needs.

## Conclusion

Notwithstanding the limitations of this review, our study provides compelling evidence for a significant association between sexual victimization and PND, independent of a history of depression and other types of abuse. These findings call attention to the dearth of existing evidence-based interventions intended to address this issue. This review also calls for future research on evidence-based interventions during the perinatal period for survivors of sexual victimization. Such efforts could include development and evaluation of integrated mental health and perinatal health care, improved and increased screening practices for a history of sexual victimization, and provider trainings on trauma-informed perinatal care.

### Implications for Research, Policy, and Practice

#### Research

- Additional longitudinal research is needed that incudes larger samples and randomization in order to establish causal links between PND and a lifetime history of sexual victimization.
- Longitudinal research should also test intervention(s) in order to gain more understanding of evidence-based programs that are most effective in reducing perinatal depression for survivors of sexual victimization.
- Research must include validated and/or standardized instruments when assessing for depression and sexual victimization.
- Foreground information about racial/ethnic identity in study samples and expand understanding of potential differences in associations between a lifetime history of sexual victimization and perinatal depression across identities.
- Research should continue identifying theoretically plausible mechanisms that link a lifetime history of sexual victimization and perinatal depression.

#### Policy

- Routine screening for a lifetime history of sexual victimization in the perinatal period. Assessing for victimization across the life course will help determine whether an accumulation of sexual victimization events influences the probability of PND.
- Policy that ensures all perinatal health providers are trained in trauma-informed care

#### Practice

- Incorporate trauma-informed care for all patients in the perinatal period.
- Imbed social workers into perinatal health care in order to provide comprehensive care for survivors during the perinatal period.
